# Monoclinic modification of *N*-[(1,1-dimethyl­ethoxy)carbon­yl]-3-[(*R*)-prop-2-en-1-ylsulfin­yl]-(*R*)-alanine ethyl ester at 200 (1) K

**DOI:** 10.1107/S1600536809019023

**Published:** 2009-05-23

**Authors:** Suneel P. Singh, Marcus J. Verdu, Alan J. Lough, Adrian L. Schwan

**Affiliations:** a Department of Chemistry, University of Guelph, Guelph, Ontario, Canada N1G 2W1; b Department of Chemistry, University of Toronto, Toronto, Ontario, Canada M5S 3H6

## Abstract

In the monoclinic polymorph of the title compound, C_13_H_23_NO_5_S, inter­molecular N—H⋯O hydrogen bonds link mol­ecules into one-dimensional chains along [100]. The atoms of the terminal propenyl group are disordered over two sets of sites with refined occupancies of 0.69 (2) and 0.31 (2).

## Related literature

For the crystal structure of the triclinic modification of the title compound at 120 (1) K see the paper which follows: Singh *et al.* (2009 ▶). For background information on chiral sulfoxides, see: Rose *et al.* (2005 ▶); Fernandez & Khiar, (2003 ▶); Olbe *et al.*, 2003 ▶. For synthetic details, see: O’Donnell & Schwan (2003 ▶). For related crystal structures see: Allain *et al.* (1980 ▶); Nakamura *et al.* (1996 ▶). For temperature-dependent phase trans­ition in cysteine, see: Paukov *et al.* (2007 ▶), Kolesov *et al.* (2008 ▶).
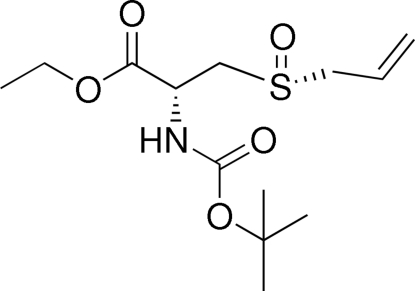


## Experimental

### 

#### Crystal data

C_13_H_23_NO_5_S
*M* *_r_* = 305.38
Monoclinic, 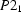

*a* = 5.1859 (6) Å
*b* = 11.5202 (18) Å
*c* = 14.009 (2) Å
β = 96.396 (8)°
*V* = 831.7 (2) Å^3^
*Z* = 2
Mo *K*α radiation
μ = 0.21 mm^−1^
*T* = 200 K
0.38 × 0.12 × 0.12 mm

#### Data collection

Nonius KappaCCD diffractometer
Absorption correction: multi-scan (*SORTAV*; Blessing, 1995 ▶) *T*_min_ = 0.711, *T*_max_ = 0.974
3908 measured reflections
2397 independent reflections
1844 reflections with *I* > 2σ(*I*)
*R*_int_ = 0.070

#### Refinement

*R*[*F* ^2^ > 2σ(*F* ^2^)] = 0.059
*wR*(*F* ^2^) = 0.164
*S* = 1.05
2397 reflections
196 parameters
4 restraints
H-atom parameters constrained
Δρ_max_ = 0.30 e Å^−3^
Δρ_min_ = −0.22 e Å^−3^
Absolute structure: Flack (1983 ▶), 898 Friedel pairs
Flack parameter: −0.08 (15)

### 

Data collection: *COLLECT* (Nonius, 2002 ▶); cell refinement: *DENZO-SMN* (Otwinowski & Minor, 1997 ▶); data reduction: *DENZO-SMN*; program(s) used to solve structure: *SIR92* (Altomare *et al*., 1994 ▶); program(s) used to refine structure: *SHELXTL* (Sheldrick, 2008 ▶); molecular graphics: *PLATON* (Spek, 2009 ▶); software used to prepare material for publication: *SHELXTL*.

## Supplementary Material

Crystal structure: contains datablocks global, I. DOI: 10.1107/S1600536809019023/hb2978sup1.cif

Structure factors: contains datablocks I. DOI: 10.1107/S1600536809019023/hb2978Isup2.hkl

Additional supplementary materials:  crystallographic information; 3D view; checkCIF report

## Figures and Tables

**Table 1:** Hydrogen-bond geometry (Å, °)

*D*—H⋯*A*	*D*—H	H⋯*A*	*D*⋯*A*	*D*—H⋯*A*
N1—H1⋯O4^i^	0.88	2.20	2.967 (5)	145

Symmetry code: (i) 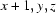.

**Fig. 1.**
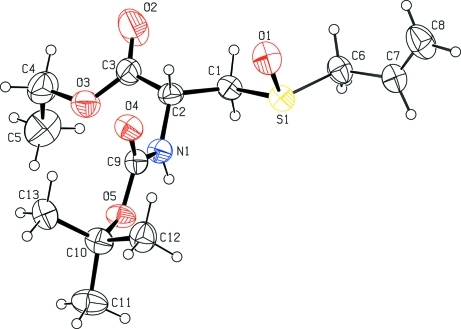
The molecular structure of (I): displacement elllipsoids are drawn at the 30% probabilty level. The minor disorder component is not shown.

**Fig. 2.**
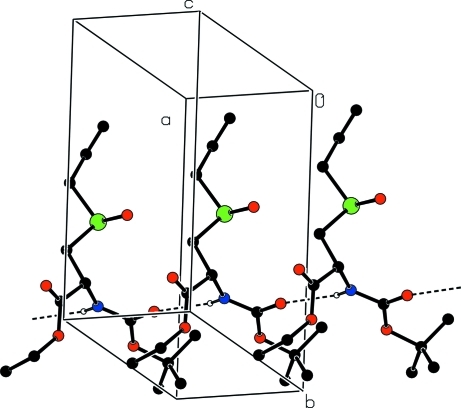
Part of the crystal structure of (I) with hydrogen bonds shown as dashed lines.

**Fig. 3.**
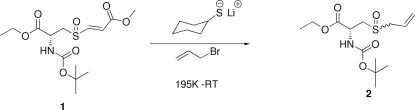
The synthetic scheme.

## supplementary crystallographic information

### Comment

A number of biologically significant molecules have a chiral sulfoxide functionality in their structure which is important for stereochemically dependent biological actions like metabolism and enzyme inhibition (Rose *et al.*, 2005). Owing to the existence of pharmacological and toxicological differences between stereoisomers, chiral recognition has now become an integral part of drug research and development. Therefore, there has been a great interest in the synthesis of optically pure sulfoxides in recent years (Fernandez & Khiar, 2003). For example, the chiral switch drug esomeprazole [(S) isomer of omeprazole], the first single-optical-isomer gastric proton pump inhibitor (PPI), generally provides better acid control than current racemic PPIs and has a favourable pharmacokinetic profile relative to omeprazole (Olbe *et al.*, 2003). However, only few examples of crystal structures of chirally pure cysteinyl sulfoxides have been reported in the literature (Nakamura *et al.*, 1996). Moreover, temperature dependent phase trasitions in the crystal structures of cysteinyl sulfoxides are unknown till date (Paukov *et al.*, 2007 and Kolesov *et al.*, 2008).

The molecular structure of the title compound, (I), is shown in Fig. 1. In the crystal structure, intermolecular N—H···O hydrogen bonds link molecules into one-dimensional chains along [100] (Table 1, Fig. 2). Data for the title compound (using the same crystal) were also collected at 120 (1) K and the crystal structure solves and refines in the triclinic space group P1 (Singh *et al.*, 2009).

### Experimental

The N-protected amino acid derivative 1 (Fig. 3) was synthesized following a reported procedure. N-Boc-Alliin-OEt 2 was also synthesized by following the described procedure for its benzyl analog (O'Donnell and Schwan, 2003). Rerystallization from ethyl acetate and hexanes gave major diastereomer (*R*_C_, *R*_S_) of 2 as white solid, which was then dissolved in ethyl acetate and hexanes (9:1) and the solvent was allowed to evaporate slowly for several days to give white crystals of the major diastereomer (*R*_C_,*R*_S_) of 2.

### Refinement

Hydrogen atoms were placed in calculated positions with C—H = 0.95–0.99; N—H = 0.88 Å and refined as riding with *U*_iso_(H) = 1.2*U*_eq_(C, N) or 1.5*U*_eq_(methyl C).  The atoms of the terminal propenyl group are disordered over two sites with refined occupancies of 0.69 (2) and 0.31 (2); the bond lengths in the minor component were restrained to be equal to those of the major component.

### Crystal data

**Table:**

C_13_H_23_NO_5_S	*F*(000) = 328
*M**_r_* = 305.38	*D*_x_ = 1.219 Mg m^−^^3^
Monoclinic, *P*2_1_	Mo *K*α radiation, λ = 0.71073 Å
Hall symbol: P 2yb	Cell parameters from 3908 reflections
*a* = 5.1859 (6) Å	θ = 2.9–25.0°
*b* = 11.5202 (18) Å	µ = 0.21 mm^−^^1^
*c* = 14.009 (2) Å	*T* = 200 K
β = 96.396 (8)°	Needle, colourless
*V* = 831.7 (2) Å^3^	0.38 × 0.12 × 0.12 mm
*Z* = 2	

### Data collection

**Table:**

Nonius KappaCCD diffractometer	2397 independent reflections
Radiation source: fine-focus sealed tube	1844 reflections with *I* > 2σ(*I*)
graphite	*R*_int_ = 0.070
Detector resolution: 9 pixels mm^-1^	θ_max_ = 25.0°, θ_min_ = 2.9°
φ scans and ω scans with κ offsets	*h* = −6→6
Absorption correction: multi-scan (*SORTAV*; Blessing, 1995)	*k* = −12→13
*T*_min_ = 0.711, *T*_max_ = 0.974	*l* = −14→16
3908 measured reflections	

### Refinement

**Table:**

Refinement on *F*^2^	Secondary atom site location: difference Fourier map
Least-squares matrix: full	Hydrogen site location: inferred from neighbouring sites
*R*[*F*^2^ > 2σ(*F*^2^)] = 0.059	H-atom parameters constrained
*wR*(*F*^2^) = 0.164	*w* = 1/[σ^2^(*F*_o_^2^) + (0.0816*P*)^2^ + 0.1385*P*] where *P* = (*F*_o_^2^ + 2*F*_c_^2^)/3
*S* = 1.05	(Δ/σ)_max_ = 0.002
2397 reflections	Δρ_max_ = 0.30 e Å^−^^3^
196 parameters	Δρ_min_ = −0.22 e Å^−^^3^
4 restraints	Absolute structure: Flack (1983), 898 Friedel pairs
Primary atom site location: structure-invariant direct methods	Flack parameter: −0.08 (15)

### Special details

**Table:**

Experimental. Absorption correction: multi-scan from symmetry-related measurements (SORTAV; Blessing, 1995)
Geometry. All e.s.d.'s (except the e.s.d. in the dihedral angle between two l.s. planes) are estimated using the full covariance matrix. The cell e.s.d.'s are taken into account individually in the estimation of e.s.d.'s in distances, angles and torsion angles; correlations between e.s.d.'s in cell parameters are only used when they are defined by crystal symmetry. An approximate (isotropic) treatment of cell e.s.d.'s is used for estimating e.s.d.'s involving l.s. planes.
Refinement. Refinement of *F*^2^ against ALL reflections. The weighted *R*-factor *wR* and goodness of fit *S* are based on *F*^2^, conventional *R*-factors *R* are based on *F*, with *F* set to zero for negative *F*^2^. The threshold expression of *F*^2^ > σ(*F*^2^) is used only for calculating *R*-factors(gt) *etc*. and is not relevant to the choice of reflections for refinement. *R*-factors based on *F*^2^ are statistically about twice as large as those based on *F*, and *R*- factors based on ALL data will be even larger.

### Fractional atomic coordinates and isotropic or equivalent isotropic displacement parameters (Å^2^)

**Table:**

	*x*	*y*	*z*	*U*_iso_*/*U*_eq_	Occ. (<1)
S1	0.4365 (2)	0.46507 (13)	0.25026 (8)	0.0632 (4)	
O1	0.1763 (5)	0.4581 (4)	0.2851 (3)	0.0810 (10)	
O2	0.6209 (19)	0.7332 (5)	0.4815 (4)	0.175 (3)	
O3	0.6089 (8)	0.8717 (4)	0.3748 (3)	0.0850 (11)	
O4	0.0034 (6)	0.7655 (3)	0.2141 (2)	0.0667 (9)	
O5	0.2554 (5)	0.8446 (3)	0.1080 (2)	0.0656 (9)	
N1	0.4391 (7)	0.7397 (4)	0.2270 (3)	0.0576 (10)	
H1	0.5784	0.7447	0.1966	0.069*	
C1	0.6118 (9)	0.5732 (4)	0.3246 (4)	0.0578 (12)	
H1A	0.6416	0.5449	0.3916	0.069*	
H1B	0.7828	0.5871	0.3015	0.069*	
C2	0.4583 (9)	0.6867 (4)	0.3214 (3)	0.0594 (12)	
H2A	0.2780	0.6674	0.3351	0.071*	
C3	0.5757 (12)	0.7652 (5)	0.4014 (4)	0.0775 (16)	
C4	0.7307 (14)	0.9553 (8)	0.4454 (5)	0.1019 (19)	
H4A	0.6164	1.0237	0.4490	0.122*	
H4B	0.7574	0.9189	0.5097	0.122*	
C5	0.9780 (13)	0.9914 (8)	0.4162 (6)	0.116 (3)	
H5A	1.0601	1.0474	0.4627	0.174*	
H5B	0.9502	1.0276	0.3526	0.174*	
H5C	1.0909	0.9235	0.4135	0.174*	
C6	0.628 (4)	0.3423 (8)	0.2976 (17)	0.067 (5)	0.694 (18)
H6A	0.8146	0.3592	0.2950	0.080*	0.694 (18)
H6B	0.6012	0.3302	0.3657	0.080*	0.694 (18)
C7	0.557 (2)	0.2340 (8)	0.2422 (9)	0.082 (3)	0.694 (18)
H7	0.6299	0.2247	0.1833	0.098*	0.694 (18)
C8	0.406 (2)	0.1516 (9)	0.2657 (9)	0.100 (4)	0.694 (18)
H8A	0.3285	0.1565	0.3238	0.120*	0.694 (18)
H8B	0.3740	0.0861	0.2249	0.120*	0.694 (18)
C6A	0.600 (8)	0.3431 (15)	0.312 (3)	0.057 (13)*	0.306 (18)
H6A1	0.7852	0.3428	0.3015	0.069*	0.306 (18)
H6A2	0.5896	0.3502	0.3824	0.069*	0.306 (18)
C7A	0.474 (5)	0.233 (2)	0.2760 (18)	0.076 (9)*	0.306 (18)
H7A	0.3078	0.2138	0.2940	0.091*	0.306 (18)
C8A	0.582 (6)	0.161 (3)	0.2209 (17)	0.100 (9)*	0.306 (18)
H8A1	0.7483	0.1786	0.2021	0.120*	0.306 (18)
H8A2	0.4950	0.0921	0.1994	0.120*	0.306 (18)
C9	0.2142 (8)	0.7809 (4)	0.1855 (3)	0.0542 (11)	
C10	0.0367 (8)	0.8905 (5)	0.0423 (4)	0.0668 (14)	
C11	0.1753 (10)	0.9514 (7)	−0.0349 (4)	0.0905 (18)	
H11A	0.2817	1.0150	−0.0056	0.136*	
H11C	0.0462	0.9825	−0.0848	0.136*	
H11D	0.2866	0.8956	−0.0637	0.136*	
C12	−0.1300 (11)	0.7915 (5)	−0.0014 (4)	0.0801 (16)	
H12C	−0.2007	0.7478	0.0497	0.120*	
H12D	−0.0241	0.7399	−0.0367	0.120*	
H12A	−0.2729	0.8229	−0.0456	0.120*	
C13	−0.1142 (9)	0.9761 (5)	0.0933 (4)	0.0732 (13)	
H13C	−0.2131	0.9352	0.1386	0.110*	
H13D	−0.2339	1.0182	0.0465	0.110*	
H13A	0.0053	1.0312	0.1284	0.110*	

### Atomic displacement parameters (Å^2^)

**Table:**

	*U*^11^	*U*^22^	*U*^33^	*U*^12^	*U*^13^	*U*^23^
S1	0.0581 (6)	0.0632 (7)	0.0675 (7)	−0.0011 (7)	0.0036 (5)	0.0038 (7)
O1	0.0487 (16)	0.079 (2)	0.114 (3)	−0.005 (2)	0.0032 (16)	0.000 (3)
O2	0.370 (10)	0.077 (3)	0.064 (3)	0.004 (5)	−0.035 (4)	−0.003 (3)
O3	0.108 (3)	0.077 (3)	0.070 (2)	−0.019 (2)	0.0107 (19)	−0.002 (2)
O4	0.0447 (16)	0.076 (2)	0.080 (2)	0.0025 (17)	0.0113 (15)	0.0132 (17)
O5	0.0517 (17)	0.083 (2)	0.062 (2)	0.0055 (17)	0.0057 (13)	0.0213 (18)
N1	0.044 (2)	0.065 (3)	0.064 (2)	0.0082 (19)	0.0095 (16)	0.006 (2)
C1	0.052 (2)	0.059 (3)	0.062 (3)	−0.004 (2)	0.004 (2)	0.004 (2)
C2	0.057 (3)	0.066 (3)	0.056 (3)	0.006 (2)	0.014 (2)	0.008 (2)
C3	0.102 (4)	0.067 (4)	0.063 (4)	0.022 (3)	0.003 (3)	0.004 (3)
C4	0.116 (5)	0.100 (5)	0.090 (4)	−0.014 (5)	0.011 (3)	−0.023 (4)
C5	0.089 (4)	0.140 (8)	0.115 (5)	−0.022 (5)	−0.005 (4)	−0.028 (5)
C6	0.056 (6)	0.058 (7)	0.085 (9)	−0.004 (4)	0.005 (8)	0.005 (4)
C7	0.100 (8)	0.062 (7)	0.084 (7)	0.010 (6)	0.015 (6)	0.005 (6)
C8	0.117 (9)	0.071 (7)	0.105 (8)	−0.017 (6)	−0.021 (6)	0.007 (6)
C9	0.047 (3)	0.054 (3)	0.061 (3)	0.001 (2)	0.004 (2)	−0.002 (2)
C10	0.049 (2)	0.078 (4)	0.072 (3)	0.008 (3)	0.001 (2)	0.014 (3)
C11	0.075 (3)	0.123 (5)	0.073 (3)	0.005 (4)	0.002 (2)	0.033 (4)
C12	0.079 (3)	0.078 (4)	0.081 (4)	0.012 (3)	−0.003 (3)	−0.004 (3)
C13	0.064 (3)	0.061 (3)	0.092 (3)	−0.001 (3)	−0.002 (2)	0.003 (3)

### Geometric parameters (Å, °)

**Table:**

S1—O1	1.487 (3)	C6—H6A	0.9900
S1—C1	1.803 (5)	C6—H6B	0.9900
S1—C6	1.813 (6)	C7—C8	1.296 (12)
S1—C6A	1.813 (7)	C7—H7	0.9500
O2—C3	1.179 (7)	C8—H8A	0.9500
O3—C3	1.300 (7)	C8—H8B	0.9500
O3—C4	1.471 (8)	C6A—C7A	1.493 (14)
O4—C9	1.218 (5)	C6A—H6A1	0.9900
O5—C9	1.347 (6)	C6A—H6A2	0.9900
O5—C10	1.477 (5)	C7A—C8A	1.296 (13)
N1—C9	1.331 (6)	C7A—H7A	0.9500
N1—C2	1.450 (6)	C8A—H8A1	0.9500
N1—H1	0.8800	C8A—H8A2	0.9500
C1—C2	1.529 (7)	C10—C13	1.491 (8)
C1—H1A	0.9900	C10—C12	1.518 (8)
C1—H1B	0.9900	C10—C11	1.534 (8)
C2—C3	1.514 (8)	C11—H11A	0.9800
C2—H2A	1.0000	C11—H11C	0.9800
C4—C5	1.450 (10)	C11—H11D	0.9800
C4—H4A	0.9900	C12—H12C	0.9800
C4—H4B	0.9900	C12—H12D	0.9800
C5—H5A	0.9800	C12—H12A	0.9800
C5—H5B	0.9800	C13—H13C	0.9800
C5—H5C	0.9800	C13—H13D	0.9800
C6—C7	1.494 (13)	C13—H13A	0.9800
			
O1—S1—C1	105.4 (2)	C8—C7—H7	116.5
O1—S1—C6	108.6 (9)	C6—C7—H7	116.5
C1—S1—C6	96.2 (4)	C7—C8—H8A	120.0
O1—S1—C6A	101.1 (18)	C7—C8—H8B	120.0
C1—S1—C6A	94.6 (9)	H8A—C8—H8B	120.0
C3—O3—C4	119.1 (5)	C7A—C6A—S1	109.5 (16)
C9—O5—C10	121.2 (3)	C7A—C6A—H6A1	109.8
C9—N1—C2	121.1 (4)	S1—C6A—H6A1	109.8
C9—N1—H1	119.5	C7A—C6A—H6A2	109.8
C2—N1—H1	119.5	S1—C6A—H6A2	109.8
C2—C1—S1	110.3 (3)	H6A1—C6A—H6A2	108.2
C2—C1—H1A	109.6	C8A—C7A—C6A	123 (4)
S1—C1—H1A	109.6	C8A—C7A—H7A	118.6
C2—C1—H1B	109.6	C6A—C7A—H7A	118.6
S1—C1—H1B	109.6	C7A—C8A—H8A1	120.0
H1A—C1—H1B	108.1	C7A—C8A—H8A2	120.0
N1—C2—C3	113.9 (4)	H8A1—C8A—H8A2	120.0
N1—C2—C1	111.7 (4)	O4—C9—N1	125.4 (4)
C3—C2—C1	108.9 (4)	O4—C9—O5	125.0 (4)
N1—C2—H2A	107.4	N1—C9—O5	109.6 (4)
C3—C2—H2A	107.4	O5—C10—C13	110.2 (4)
C1—C2—H2A	107.4	O5—C10—C12	110.3 (4)
O2—C3—O3	123.3 (6)	C13—C10—C12	112.6 (4)
O2—C3—C2	122.6 (6)	O5—C10—C11	102.5 (3)
O3—C3—C2	114.0 (5)	C13—C10—C11	110.3 (5)
C5—C4—O3	109.0 (5)	C12—C10—C11	110.5 (5)
C5—C4—H4A	109.9	C10—C11—H11A	109.5
O3—C4—H4A	109.9	C10—C11—H11C	109.5
C5—C4—H4B	109.9	H11A—C11—H11C	109.5
O3—C4—H4B	109.9	C10—C11—H11D	109.5
H4A—C4—H4B	108.3	H11A—C11—H11D	109.5
C4—C5—H5A	109.5	H11C—C11—H11D	109.5
C4—C5—H5B	109.5	C10—C12—H12C	109.5
H5A—C5—H5B	109.5	C10—C12—H12D	109.5
C4—C5—H5C	109.5	H12C—C12—H12D	109.5
H5A—C5—H5C	109.5	C10—C12—H12A	109.5
H5B—C5—H5C	109.5	H12C—C12—H12A	109.5
C7—C6—S1	111.5 (7)	H12D—C12—H12A	109.5
C7—C6—H6A	109.3	C10—C13—H13C	109.5
S1—C6—H6A	109.3	C10—C13—H13D	109.5
C7—C6—H6B	109.3	H13C—C13—H13D	109.5
S1—C6—H6B	109.3	C10—C13—H13A	109.5
H6A—C6—H6B	108.0	H13C—C13—H13A	109.5
C8—C7—C6	126.9 (18)	H13D—C13—H13A	109.5

### Hydrogen-bond geometry (Å, °)

**Table:**

*D*—H···*A*	*D*—H	H···*A*	*D*···*A*	*D*—H···*A*
N1—H1···O4^i^	0.88	2.20	2.967 (5)	145

Symmetry codes: (i) *x*+1, *y*, *z*.
